# Supplementary material for: Association between oral microbiome and seven types of cancers in East Asian population: a two-sample Mendelian randomization analysis
Source: Front Mol Biosci. 2023 Nov 21;10:1327893. doi: 10.3389/fmolb.2023.1327893 (PMC10702768; doi:10.3389/fmolb.2023.1327893)
Supplement: Supplementary file 1 [file DataSheet1.ZIP › Supplementary Materials/MR plots for tongue/tongue═╝/Gastric cancer/pheno.1392_to_gastric cancer_forest.pdf]

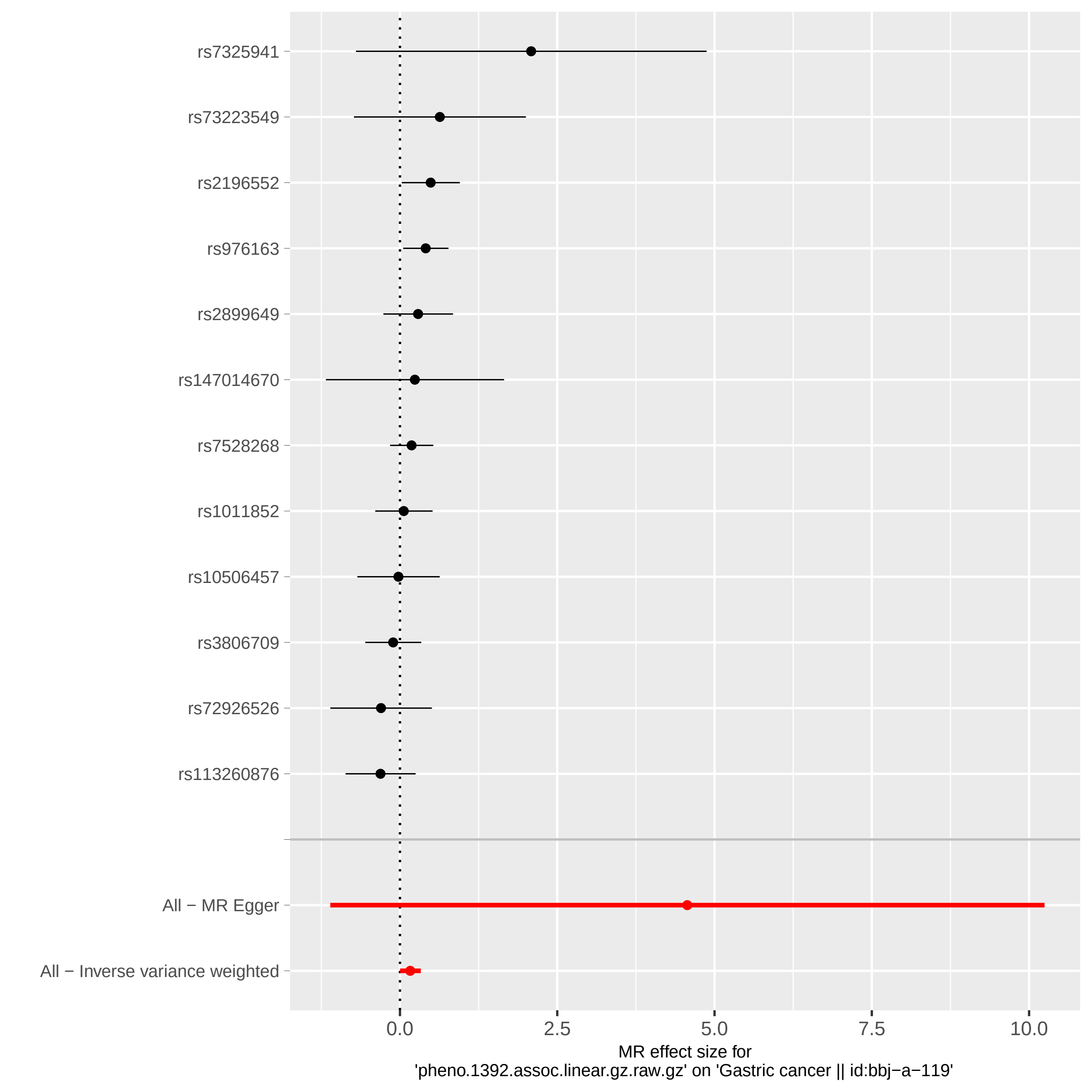
rs7325941
rs73223549
rs2196552
rs976163
rs2899649
rs147014670
rs7528268
rs1011852
rs10506457
rs3806709
rs72926526
rs113260876
All − MR Egger
All − Inverse variance weighted
0.0
2.5
5.0
7.5
10.0
MR effect size for
'pheno.1392.assoc.linear.gz.raw.gz' on 'Gastric cancer || id:bbj−a−119'
